# The nasopharyngeal microbiota of feedlot cattle

**DOI:** 10.1038/srep15557

**Published:** 2015-10-26

**Authors:** Devin B. Holman, Edouard Timsit, Trevor W. Alexander

**Affiliations:** 1 Lethbridge Research Centre, Agriculture and Agri-Food Canada, Lethbridge, AB, Canada; 2 Department of Production Animal Health, Faculty of Veterinary Medicine, University of Calgary, Calgary, AB, Canada

## Abstract

The bovine nasopharyngeal tract plays an important role in animal health and welfare by acting as a site for the carriage of pathogens causing bovine respiratory disease, a condition which results in significant morbidity and mortality in feedlot cattle. We characterized the bacterial nasopharyngeal microbiota in cattle at feedlot entry (day 0) and day 60 using 454 pyrosequencing. We also identified the most frequently isolated aerobic bacteria from nasopharyngeal swabs after plating onto three types of media. The cattle nasopharyngeal microbiota was composed primarily of *Proteobacteria* (68.9%) and *Firmicutes* (19.2%). At the genus-level, there was more inter-individual variability and a total of 55 genera were identified. The genera *Pseudomonas* (23.7%), *Shewanella* (23.5%), *Acinetobacter* (17.5%), and *Carnobacterium* (12.2%) were most prevalent at entry, while after 60 days in the feedlot, *Staphylococcus* (20.8%), *Mycoplasma* (14.9%), *Mannheimia* (10.4%), and *Moraxella* (9.4%) were dominant. The nasopharyngeal microbiota also became more homogenous after 60 days in the feedlot and differed in structure at day 0 and 60. Using culture-based methods, the most frequently isolated bacteria from nasopharyngeal swabs were *Bacillus*, *Staphylococcus*, *Moraxella*, *Pasteurella*, and *Mannheimia*. These results provide insight into the nasopharyngeal microbiota of cattle and demonstrate that specific changes take place during feedlot production.

The bovine nasopharynx serves as a niche for opportunistic bacterial pathogens and also a site of entry for infectious agents that can cause bovine respiratory disease (BRD^1^). In the United States, the most recent survey indicated that 16.2% of all feedlot cattle were diagnosed with BRD at some point during production and that 96.9% of all large feedlots (>1000 head cattle) were affected^1^. Consequently, it is the most common reason for treatment and the most expensive disease to treat in feedlot cattle. It is also a significant cause of mortality as BRD is fatal in 4% of cattle treated for BRD^1^.

The primary treatment for cattle diagnosed with BRD is an injectable antimicrobial. In the United States 100% of large feedlots use injectable antibiotics to treat BRD and in Canada 20 to 50% of cattle receive an injectable antimicrobial upon entry to the feedlot as a metaphylactic measure to control BRD^1^,^2^. However, the use of antimicrobial agents in cattle can select for antimicrobial-resistant bacteria^3^,^4^. The development of mitigation strategies (e.g. probiotics and prebiotics) to reduce antimicrobial use in treating BRD would be greatly aided with the characterization of the nasopharyngeal tract microbiota in cattle. Presently, studies involving the nasopharyngeal tract microbiota have been mostly limited to the pathogenic bacteria associated with BRD such as *Mannheimia haemolytica*, *Mycoplasma bovis*, *Pasteurella multocida*, and *Histophilus somni*^5^.

The objective of the current study was therefore to characterize the nasopharyngeal tract microbiota of feedlot cattle at entry (day 0) and after 60 days in the feedlot using high throughput sequencing. This culture-independent approach was complemented with culture-based methods for comparison.

## Results

### Analysis of 16S rRNA sequences from cultured bacteria

Aerobic bacteria from nasopharyngeal swabs taken from steers at entry and 60 days after feedlot placement were cultivated on three different media: brain heart infusion (BHI), 5% sheep blood, and de Man, Rogosa and Sharpe (MRS) agar. In total, 605 bacterial isolates were selected for sequencing (Tables 1 and 2). Using near full-length 16S rRNA gene sequences from each isolate and BLAST analysis, 32 different genera were identified across all samples using the three different media. On blood and BHI media, the most frequently isolated bacteria were members of the phyla *Firmicutes* and *Proteobacteria* (Table 1). The genera *Moraxella*, *Pasteurella*, *Mannheimia*, *Corynebacterium* and *Acinetobacter* accounted for the majority of bacteria isolated on these media and all were detected at both feedlot entry and after 60 days (Table 1). Notably, bacteria belonging to the genera *Mannheimia* and *Pasteurella* comprised greater than 7% of the isolates at both day 0 and day 60. Two species of these genera, *P. multocida* and *M. haemolytica*, are often isolated from cattle suffering from BRD^6^. The most abundant genera observed were cultured from both BHI and blood media. Certain genera however, such as *Aerococcus* and *Psychrobacter* were isolated only from a single medium.

There was considerably less bacterial growth on MRS agar compared to the BHI and blood agars. In some instances, no colonies were observed from nasopharyngeal samples plated on MRS agar. This was expected, given that MRS agar is the most selective of the three media used. For this reason, all isolates observed were subcultured and sequenced. The majority of bacteria from MRS media were from the phylum *Firmicutes* and the *Bacillus* and *Staphylococcus* genera (Table 2). Four genera (*Lactobacillus*, *Enterococcus*, *Rummeliibacillus*, and *Pediococcus*) that were isolated from MRS agar were not observed on the BHI and blood media.

### Analysis of 16S rRNA sequences from metagenomic DNA

A total of 51,118 sequences with an average length of 457 bp remained following quality-filtering and chimera removal. These sequences were clustered into OTUs (97%) using the *de novo* method and the removal of singletons resulted in a total of 864 OTUs. All samples were rarefied to 3000 sequences per sample to account for uneven sequencing depth. Good’s coverage was 97% for all samples indicating that most of the OTUs were captured during sequencing.

**Figure 1:**
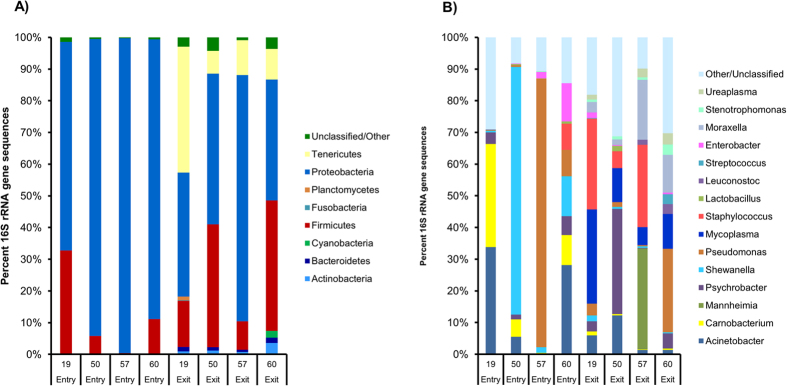
Classification of 16S rRNA gene sequences at the (**A**) phylum and (**B**) genera level for each individual day 0 and day 60 nasopharyngeal sample. Numbers on the x-axis indicate animal identification number. All phyla are shown in (**A**) and in (**B**) only those genera comprising greater than 1% of the sequences at either entry or exit are displayed.

Taxonomy was assigned to OTUs using the RDP classifier and the SILVA database (Fig. 1). A total of eight bacterial phyla were identified during the study although four of these phyla were only found in individual steers after 60 days in the feedlot. At entry into the feedlot the large majority of sequences were classified as *Proteobacteria*. In two entry samples, *Proteobacteria* comprised greater than 93% of all sequences while at day 60, only one steer had greater than 50% *Proteobacteria* in their nasopharyngeal tract. At feedlot entry, *Firmicutes* was the only other phylum with more than 1% of the sequences, while at day 60, *Actinobacteria*, *Tenericutes*, and *Bacteroidetes* were found at a relative abundance of greater than 1% of sequences. Overall, only 1.8% of sequences could not be classified at the phylum-level (Fig. 1A).

The sequences were assigned to 55 different genera with 15 of these comprising greater than 1% of sequences at either day 0 or day 60 (Fig. 1B). Overall, 15.8% of the sequences from entry samples and 13.1% of sequences from day 60 samples could not be classified at the genus-level. The dominate genera were very different between the two sampling points. While *Staphylococcus*, *Mycoplasma*, *Mannheimia*, and *Moraxella* were the four most prevalent genera in day 60 samples, they accounted for less than 0.1% of the sequences in entry samples. The four most dominate genera in entry samples, *Pseudomonas*, *Shewanella*, *Acinetobacter*, and *Carnobacterium*, were also found in day 60 samples although at a much lower abundance (Fig. 1B).

**Figure 2:**
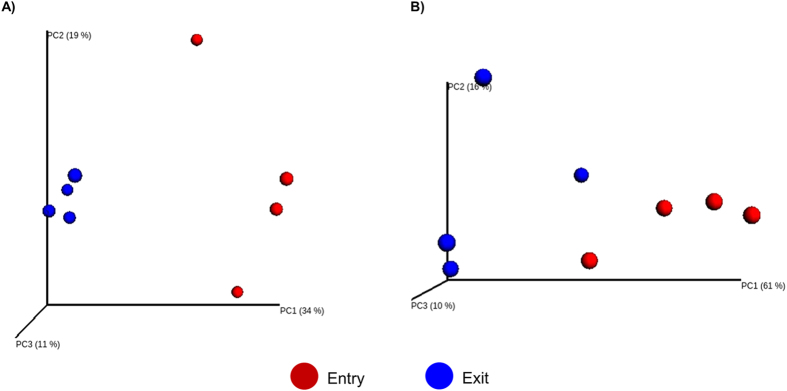
Principal coordinate analysis (PCoA) of the (**A**) unweighted UniFrac distances and (**B**) weighted UniFrac distances for day 0 and day 60 nasopharyngeal samples. The percent variation explained by each principal coordinate is indicated on the axes.

The alpha diversity of the cattle nasopharyngeal samples was assessed using several metrics (Table 3). None of the alpha diversity measures differed between day 0 and day 60 feedlot samples (p < 0.05), although the day 0 nasopharyngeal samples were more diverse and had a greater number of OTUs. The beta diversity was measured using the phylogeny-based unweighted and weighted UniFrac distances and visualized using PCoA plots (Fig. 2). When the unweighted and weighted UniFrac distances for nasopharyngeal day 0 and day 60 samples were compared using ANOSIM, the two time points were different (p < 0.05) from one another and the associated R-value was relatively large for both unweighted (0.833) and weighted (0.677) UniFrac indicating that the two time points are separated from each other.

The core microbiota of the nasopharyngeal samples was also determined. The core microbiota can be defined as those OTUs found in all samples at a particular time point. Including both entry and day 60 samples together, only one OTU was common to all samples, which was classified as belonging to the *Carnobacterium* genus. Entry samples shared nine OTUs with each other, with all but one belonging to the *Proteobacteria* phylum (Supplementary Table S1). The core microbiota of the day 60 nasopharyngeal samples was comprised of 16 OTUs, four of which were classified at the genus-level as *Staphylococcus* and two each as *Pseudomonas* and *Mycoplasma* (Supplementary Table S2). When the relative abundance of OTUs was compared between entry and day 60 nasopharyngeal samples using the G-test, 34 OTUs were found to be differentially abundant between the two time points (FDR < 0.05; Supplementary Table S3). Of these differentially abundant OTUs, 29 were more abundant in the day 0 samples and five in the day 60 samples. Not surprisingly given the relative abundance of *Proteobacteria* at entry, all but one of the OTUs that were more abundant at this time point belonged to this phylum.

## Discussion

In this study we describe for the first time the nasopharyngeal microbiota of feedlot steers at two different time points, entry and after 60 days in the feedlot, using high throughput sequencing. In addition, we used culture-based techniques to assess the cultivable fraction. The bovine nasopharyngeal tract is of particular interest as respiratory infections (i.e. BRD) are the leading cause of morbidity and mortality in post-weaned feedlot cattle in North America^7^,^8^. We selected the time point 60 days for analysis because the majority of BRD mortalities occur within the first 60 days of feedlot placement^6^, suggesting that the microbiota within this period may be important to cattle health.

It has been estimated that perhaps 1% of bacteria in some ecological niches such as the mammalian intestine are culturable in the laboratory^9^. A comparison of the 454 pyrosequencing and culture-based analysis from these same animals highlights some of the limitations of culture-dependent analysis. For example, the *Bacillus* genus was cultured frequently from the nasopharynx of the steers on all three media yet *Bacillus* was identified in only one steer at day 60 when analyzed using 454 pyrosequencing and at a relative abundance of less than 0.1%, indicating that culturing over-represented this genus. The same is true for the genus *Pasteurella* which was found only in two of day 60 samples, at levels of less than 0.05% of sequences, while culturing on non-selective media indicated that 17.1% of isolated bacteria belonged to this genus. At the same time, several of the most frequently isolated bacterial genera such as *Acinetobacter*, *Moraxella*, *Mannheimia*, *Pyschrobacter*, *Staphylococcus*, and *Streptococcus* were also among the most commonly identified sequences in the 454 pyrosequencing samples (Tables 1 and 2; Fig. 1). The *Proteobacteria* and *Firmicutes* phyla also dominated both the culture-based and culture-independent analyses.

Overall, while 32 genera could be isolated using culture-based methods, 454 pyrosequencing revealed that at least 55 genera were present in the nasopharynx of the steers. These results indicate that while the culture media used in this study can identity some of the major bacterial taxa, it can also produce a limited view of the diversity of the microbiota and miss some of the more difficult to culture, yet prevalent bacteria, such as *Mycoplasma* and *Ureaplasma*^10^. Interestingly though, the *Aerococcus*, *Dietzia*, *Proteus, Rothia*, and *Microccoccus/Macrococcus* genera were identified using culturing but were not among the 454 sequences analyzed in these same steers (data not shown; Fig. 1A). This finding is a well-documented phenomenon in studies that compare culture vs. molecular-based methods^11^,^12^,^13^. It is possible that bacterial genera identified using culturing but not with pyrosequencing were at a very low abundance in the samples or that greater sequencing depth was necessary to identify them. Other technical reasons for this discrepancy could be due to the primer pair used, the DNA extraction procedure, or the sequences could be among those that could not be assigned taxonomy by the RDP classifier. Nonetheless, all of the most frequently isolated genera identified through culturing were also identified using pyrosequencing.

The dominant phylum in the nasopharynx of all cattle was the *Proteobacteria*, a finding that is consistent with studies characterizing the nasopharyngeal tract of swine^14^, cats, dogs^15^, and even in human infants^16^. Correspondingly, the most abundant genera in entry nasopharyngeal samples were members of this phylum (Fig. 1B). At day 60, genera from the *Firmicutes* and *Tenericutes* phyla were also prevalent. During their time in the feedlot the emergence of *Mycoplasma*, *Mannheimia*, *Staphylococcus*, and *Moraxella* was particularly striking as these genera were found almost exclusively in the day 60 samples. The relatively high abundance of *Mycoplasma* in all day 60 nasopharyngeal samples is notable as certain *Mycoplasma* species, e.g. *Mycoplasma bovis* and *Mycoplasma arginini*, are associated with the BRD complex in feedlot cattle^17^,^18^. These data indicate that feedlots may act as a reservoir of *Mycolplasma* or as an environment that can favour colonization and growth in the upper respiratory tract.

Another finding of particular clinical importance was the observation that 41.5% of all 16S rRNA gene sequences in one steer at day 60 belonged to the genus *Mannheimia*. The species *Mannheimia haemolytica* is often isolated from fatal BRD cases and is considered the leading cause of BRD in feedlot cattle^6^,^19^. In addition, 10.6% of the bacterial isolates on non-selective media were identified as *Mannheimia* spp. (Table 1). It is generally thought that rapid growth of BRD pathogens in the upper respiratory tract predisposes to BRD. Despite these findings, none of the cattle were treated for BRD throughout the 60 day period. While it is possible that BRD cases were missed by the feedlot staff, these data support the colonization of healthy cattle by BRD pathogens and the importance of factors other than pathogen prevalence for BRD onset^5^,^20^,^21^. It should be noted however that a limitation of 16S rRNA gene analysis is the inability to identify strain virulence or serotype. For *M. haemolytica*, serotype 2 is most often identified in healthy cattle, and serotypes 1 and 6 are found in most BRD cases^22^.

Although *Shewanella* was the 2^nd^ most abundant genera in the entry samples, this was a result of 77.0% of the sequences in one sample being classified as *Shewanella*. It is possible that contamination during sampling or DNA sequencing might have been responsible, particularly since at day 60 the same steer had less than 1% of its sequences identified as *Shewanella*. However, despite the fact that *Shewanella* spp. are primarily considered marine and environmental bacteria^23^, they have occasionally been isolated from cattle^24^,^25^ and all samples had detectable *Shewanella* sequences in the present study. With the exception of one entry sample, all steers also had detectable *Enterobacter* sequences in their nasopharynx. While this may represent fecal contamination from the exterior portion of the nasopharynx of the steers, the relatively low abundance of sequences from the phylum *Bacteroidetes* indicates that swabs were not heavily contaminated during sampling. This conclusion is based on the fact that in cattle feces, the *Bacteroidetes* phylum typically comprises 20 to 40% of sequences^26^,^27^,^28^ and none of the samples in the current study had more than 1.75% *Bacteroidetes* sequences.

The differences observed among taxa, OTUs, and the microbial community structure between the two sampling times could be due to several factors (Figs 1. and 2; Supplementary Table S3). Steers sampled at day 0 had recently arrived at the feedlot and stress related to transport is known to influence bacteria in the bovine respiratory tract^29^. The steers in the current study also had been fed a backgrounding diet prior to arrival and were transitioned to a feedlot finishing diet. This alteration in diet together with the changes in their environment may have affected the nasopharyngeal microbiota. In addition, the age of the steers may have had a significant role in the composition of the nasopharyngeal microbiota as has been demonstrated in the upper respiratory tract of mice^30^.

As expected, the microbial diversity of the bovine nasopharyngeal tract was much lower than other locations such as the bovine gut^26^, rumen^31^, and in uterine fluid^32^. Despite this, a large variety of taxa and OTUs were observed in the present study. As a measure of the alpha diversity in the nasopharyngeal microbiota of the steers, the Shannon index (Table 3) was similar to that reported for the upper respiratory tract in mice (3.52–3.83)^30^ and the human adult nasopharyngeal microbiota (1.8 to 4.0)^33^. The PD whole tree values for the bovine nasopharyngeal microbiota were also comparable to those reported for the human adult nasopharyngeal tract (<6.0)^33^.

The day 60 samples tended to cluster together when unweighted UniFrac distances were calculated and visualized using PCoA (Fig. 2A). The entry samples did not cluster as closely together but were still well separated from the day 60 samples. When the weighted UniFrac distances were analyzed (Fig. 2B), the time points were still significantly different but they were not as well separated from one another. This is related to the fact that there was more variability in the relative abundance of several taxa in the entry samples compared to the day 60 samples (Fig. 1B). Although inter-individual variability still existed among the day 60 samples, the nasopharyngeal microbiota of the steers tended to become more homogenous during their time in the feedlot.

## Conclusions

The nasopharyngeal microbiota of feedlot cattle is diverse but dominated by two phyla, *Proteobacteria* and *Firmicutes*. Significant changes occurred in the community membership and structure of the nasopharyngeal microbiota of the steers after 60 days in the feedlot. During this period, the nasopharyngeal microbiota of the steers became more homogenous and genera of bacteria that have been associated with BRD became detectable. *Mannheimia* was highly abundant in one steer at day 60 while *Mycoplasma* constituted a significant portion of 16S rRNA gene sequences in all steers at day 60. The bovine nasopharyngeal tract also appears susceptible to being dominated by one bacterial genus as observed with *Pseudomonas* and *Shewanella* at feedlot entry. Culturing of bacteria from the nasopharyngeal swabs reflected a portion of the microbial diversity but as expected, this technique was biased towards certain groups of bacteria.

## Methods

### Animal husbandry and experimental design

Seventy crossbred steers (initial body weight 495 ± 37.9 kg) were blocked by weight and then randomly assigned to seven pens at the Lethbridge Research Centre feedlot (Lethbridge, Alberta, Canada). Steers were purchased from local auction markets and were fed diets typical of feedlots in Western Canada. Steers were gradually adapted from a silage-based diet (70% barley silage, 25% barley grain, and 5% mineral and vitamin supplement; dry matter basis) to a barley grain-based finishing diet (85% barley, 10% barley silage, and 5% supplement; dry matter basis) over the first 30 days in the feedlot, and remained on the finishing diet from day 30 onwards. Rations were prepared daily using a feed mixer and offered ad libitum with a daily minimum of 5 – 10% orts in each feed bunk. Cattle were cared for according to the guidelines set by the Canadian Council on Animal Care^34^. All experimental protocols using cattle were approved by the Animal Care Committee of the Lethbridge Research Centre (protocol #1227). All cattle remained healthy during the course of the study and were not treated for BRD nor did they receive any antimicrobial agents. For 454 pyrosequencing, four steers were randomly selected and sampled at entry (day 0) and 60 days after feedlot placement.

### Isolation of bacteria from nasopharyngeal samples

Nasopharyngeal samples were collected from each animal at entry (day 0) and then 60 days after feedlot placement, according to Alexander *et al.*^4^ Double-guarded swabs were used for sampling and were transported to the lab on ice for processing, within 1 hour of collection. The end of each swab was removed and vortexed in 0.7 ml brain heart infusion (BHI) broth containing 20% glycerol and 0.1 ml of serial 10-fold dilutions were spread onto BHI, MRS, or 5% sheep blood agar. All agar plates were incubated at 37 °C for 24–48 h in a 5% CO_2_-enriched environment. Up to seven randomly selected bacterial isolates were chosen from each plate and subcultured onto fresh agar plates. The same individuals were responsible for picking all the colonies at both time points. Subcultured isolates were transferred to 50 μl of Tris-EDTA (TE) buffer (Sigma Aldrich, St. Louis, MO, USA) and kept at −80 °C until use. The remaining swab-BHI mixtures were also stored at −80 °C.

### Identification of nasopharyngeal isolates

Randomly selected isolates from blood (n = 188) and BHI (n = 177) plates were selected for sequencing. Growth on MRS plates was limited and therefore all isolates observed (n = 240) were analyzed. Bacterial cells in TE buffer were lysed at 98 °C for 15 min with agitation at 300 rpm. For isolates that did not lyse by application of heat, the Soil Powerlyzer kit (MoBio Laboratories Inc., Carlsbad, CA, USA) was used to extract DNA, according to manufacturer protocols. The near full length 16S rRNA gene (>1400 bp) was amplified using the universal bacterial primers 27F (5′-AGRGTTTGATCMTGGCTCAG)^35^ and 1492R (5′-GGTTACCTTGTTACGACTT)^36^. Each PCR reaction consisted of: 1x Herculase II Fusion polymerase (Agilent Technologies, Santa Clara, CA), 0.4 μM of each primer (Eurofins MWG Operon LLC, Huntsville, AL, USA), 2 μl of the lysed bacterial solution as template, and sterile distilled water in total volume of 20 μl. The PCR conditions were as follows: an initial denaturation of 94 °C for 5 min; 35 cycles of 94 °C for 30 s, 53 °C for 45 s, 72 °C for 90 s; and a final extension at 72 °C for 10 min. PCR reactions were performed using a Bio-Rad S1000 Thermal Cycler (Bio-Rad Laboratories Ltd., Mississauga, ON, CAN). PCR products were visualized on a 0.8% (w/v) agarose gel stained with GelRed Nucleic Acid Gel Stain (Biotium Inc., Burlington, ON, CAN). PCR amplicons were sent to Eurofins MWG Operon (Huntsville, AL, USA) for sequencing using a 3730xl DNA Analyzer (Applied Biosystems, Carlsbad, California, USA). Bacterial 16S rRNA gene sequences from the nasopharyngeal isolates were manually trimmed in both directions and subjected to NCBI Basic Local Alignment Search Tool (BLAST) analysis for classification.

### DNA extraction, amplification, and 454 pyrosequencing

Total DNA was extracted from nasopharyngeal swabs using a Qiagen DNeasy Tissue kit (Qiagen Inc., Mississauga, ON, Canada), with the following modifications: cotton tips were removed from the applicators and then placed back in BHI-glycerol mixture, followed by centrifugation (13,000 × g for 5 min) to pellet the cotton and bacteria in solution. The pellets were re-suspended in 180 μl of enzymatic buffer that contained mutanolysin (300 U ml^−1^) in addition to lysozyme (20 mg ml^−1^). The mixtures were vortexed and then incubated for one hour at 37 °C. Twenty five μl of proteinase K and 200 μl Buffer AL (without ethanol) were then added, followed by vortexing and incubation at 56 °C for 30 min. Approximately 600 mg of 0.1 mm zircon/silica beads were added and mixed using a Tissue Lyser II (Qiagen) at maximum amplitude for three min. The mixtures were then centrifuged (13,000× g for 5 min), and 200 μl of ethanol was added to the supernatants, followed by vortexing. From this point, the protocol of the DNEasy Tissue Kit was followed as per manufacturer instructions.

The universal eubacterial primers 27-F (AGRGTTTGATCMTGGCTCAG) and 519-R (GWATTACCGCGGCKGCTG)^37^ were used to amplify the V1 to V3 region of the 16S rRNA gene. A unique 8-bp barcode was added to the forward primer to allow for pooling prior to sequencing. All amplification and sequencing steps were carried out at Molecular Research LP (MR DNA; Shallowater, TX, USA). Briefly, 16S rRNA gene amplicons were generated using a HotStarTaq Plus Master Mix Kit (Qiagen) with the following PCR conditions: a 3 min initial denaturation at 94 °C followed by 28 cycles of 94 °C for 30 s, 53 °C for 40 s, and 72 °C for 1 min, with a final extension of 5 min at 72 °C. Amplicons from all samples were then mixed together in equal concentrations and purified with Agencourt Ampure beads (Agencourt Bioscience Corporation, MA, USA). Amplicons were subjected to pyrosequencing on a 454 Genome Sequencer FLX Titanium system (454 Life Sciences, Roche Diagnostics Corporation, Branford, CT).

Raw 16S rRNA gene sequences were processed and analyzed using the QIIME software package v. 1.8.0^38^. Sequences were initially demultiplexed with the removal of primers and barcodes. Sequences were also subjected to quality filtering using the default parameters of the split_libraries.py command; the removal of sequences having a length <200 bp, a Phred score of <25, and homopolymer runs of >6 bp. Chimeric sequences were removed using the UCHIME algorithm^39^ implemented in USEARCH v. 6.1544^40^ and sequences were then assigned *de novo* to OTUs at 97% similarity using USEARCH. Taxonomy was assigned using the naïve Bayesian classifier and the SILVA database (v. 111) with a confidence cutoff of 0.80^41^,^42^ and sequences were aligned using PyNAST^43^. Singletons, that is those OTUs found only once in the entire dataset, were also removed prior to downstream analysis. Sequences were submitted to: NCBI Sequence Read Archive (SRA) under project accession number PRJNA275356 (http://www.ncbi.nlm.nih.gov/sra).

For subsequent alpha and beta diversity analysis, the OTU table was randomly subsampled and rarefied to 3000 sequences per sample. Alpha diversity was calculated within QIIME using the Shannon index^44^, Chao1^45^, and phylogenetic diversity (PD whole tree)^46^. Good’s coverage was also measured^47^. Alpha-diversity metrics were compared between day 0 and day 60 samples using a non-parametric t-test with 999 Monte Carlo permutations. Beta-diversity was analyzed using unweighted and weighted UniFrac^48^ and visualized as principal coordinate analysis (PCoA) plots using Emperor^49^. The unweighted and weighted UniFrac distances for day 0 and day 60 samples were compared using ANOSIM (analysis of similarities) with 999 permutations. OTU abundance was also compared between day 0 and day 60 samples using the G-test with the false discovery rate (FDR) correction^50^. All results were considered significant at P < 0.05.

## Additional Information

**How to cite this article**: Holman, D. B. *et al.* The nasopharyngeal microbiota of feedlot cattle. *Sci. Rep.* **5**, 15557; doi: 10.1038/srep15557 (2015).

## Supplementary Material

Supplementary Information

## Figures and Tables

**Table 1:** Taxonomy of bacterial isolates from nasopharynx of feedlot cattle identified by 16S rRNA sequencing and BLAST at day 0 and day 60, expressed as proportions (%) of bacterial isolates identified on Blood and BHI agar.

Phylum	Genus	Blood		BHI		Total	
		Day 0	Day 60	Day 0	Day 60	Day 0	Day 60
Proteobacteria	Moraxella	22.7	27.0	19.4	10.5	21.0	18.5
Proteobacteria	Pasteurella	11.4	13.5	18.3	24.2	14.9	19.0
Proteobacteria	Mannheimia	10.2	7.9	18.3	6.3	14.4	7.1
Actinobacteria	Corynebacterium	3.4	15.7	3.2	8.4	3.3	12.0
Proteobacteria	Acinetobacter	13.6	2.2	10.8	2.1	12.2	2.2
Firmicutes	Staphylococcus	5.7	6.7	9.7	2.1	7.7	4.3
Firmicutes	Streptococcus	2.3	3.4	6.5	11.6	4.4	7.6
Firmicutes	Bacillus	4.5	7.9	5.4	3.2	5.0	5.4
Proteobacteria	Psychrobacter	ND	ND	ND	16.8	ND	8.7
Firmicutes	Micrococcus/Macrococcus	6.8	4.5	2.2	3.2	4.4	3.8
Firmicutes	Aerococcus	8.0	2.2	ND	ND	3.9	1.1
Bacteroidetes	Sphingobacterium	4.5	ND	1.1	1.1	2.8	0.5
Actinobacteria	Dietzia	ND	4.5	ND	ND	ND	2.2
Proteobacteria	Neisseria	ND	2.2	ND	2.1	ND	2.2
Firmicutes	Exiguobacterium	1.1	ND	2.2	ND	1.7	ND
Proteobacteria	Proteus	1.1	ND	2.2	ND	1.7	ND
Actinobacteria	Actinomyces	ND	ND	ND	3.2	0.0	1.6
Actinobacteria	Sanguibacter	1.1	ND	ND	1.1	0.6	0.5
Actinobacteria	Rothia	ND	1.1	ND	1.1	ND	1.1
Actinobacteria	Arthrobacter	ND	ND	1.1	ND	0.6	ND
Proteobacteria	Enterobacter	1.1	ND	ND	ND	0.6	ND
Bacteroidetes	Wautersiella	1.1	ND	ND	ND	0.6	ND
Firmicutes	Geobacillus	1.1	ND	ND	ND	0.6	ND
Actinobacteria	Microbacterium	0.0	1.1	ND	ND	ND	0.5
Firmicutes	Brevibacillus	ND	ND	ND	1.1	ND	0.5
Actinobacteria	Tessaracoccus	ND	ND	ND	1.1	ND	0.5
Actinobacteria	Cellulomonas	ND	ND	ND	1.1	ND	0.5
	**Total number of isolates**	**88**	**89**	**93**	**95**	**181**	**184**

For each isolate, the genus with the highest identity to the 16S rRNA gene sequence is listed. ND = not detected.

**Table 2:** Taxonomy of bacterial isolates from nasopharynx of feedlot cattle identified by 16S rRNA sequencing and BLAST at day 0 and day 60, expressed as proportions (%) of bacterial isolates identified on MRS agar.

Phylum	Genus	Day 0	Day 60
Firmicutes	Bacillus	42.7	41.9
Firmicutes	Staphylococcus	33.1	41.9
Firmicutes	Lactobacillus	4.5	9.7
Firmicutes	Aerococcus	3.9	3.2
Firmicutes	Streptococcus	5.6	0.0
Firmicutes	Enterococcus	3.9	1.6
Firmicutes	Micrococcus/Macrococcus	1.1	1.6
Actinobacteria	Corynebacterium	1.7	0.0
Proteobacteria	Escherichia	1.7	0.0
Firmicutes	Rummeliibacillus	0.6	0.0
Firmicutes	Pediococcus	0.6	0.0
Proteobacteria	Moraxella	0.6	0.0
	**Total number of isolates**	**178**	**62**

For each isolate, the genus with the highest identity to the 16S rRNA gene sequence is listed. ND = not detected.

**Table 3:** Alpha diversity measures for the nasopharyngeal microbiota of steers at feedlot entry and after 60 days in the feedlot.

	PD whole tree	Chao1	Observed species	Shannon
Day 0	4.56 ± 0.67	305.92 ± 36.04	238.75 ± 34.66	3.69 ± 0.33
Day 60	5.36 ± 1.13	202.42 ± 60.98	165.75 ± 45.22	3.39 ± 0.40

n = 4 ± standard error of the mean.
