# Supplementary material for: The nasopharyngeal microbiota of feedlot cattle
Source: Sci Rep. 2015 Oct 26;5:15557. doi: 10.1038/srep15557 (PMC4620444; doi:10.1038/srep15557)
Supplement: Supplementary Information [file srep15557-s1.doc]

| **OTU** | **Taxonomy** |
| --- | --- |
| denovo1170 | Bacteria _Proteobacteria _Gammaproteobacteria __Alteromonadales __Shewanellaceae __Shewanella __uncultured_Shewanella_sp.'] |
| denovo857 | Bacteria __Proteobacteria __Gammaproteobacteria __Alteromonadales __Shewanellaceae __Shewanella'] |
| denovo272 | Bacteria __Proteobacteria __Gammaproteobacteria __Pseudomonadales __Pseudomonadaceae __Pseudomonas'] |
| denovo739 | Bacteria __Proteobacteria __Gammaproteobacteria __Alteromonadales __Shewanellaceae __Shewanella'] |
| denovo0 | Bacteria __Firmicutes __Bacilli __Lactobacillales __Carnobacteriaceae __Carnobacterium __uncultured_bacterium'] |
| denovo10 | Bacteria __Proteobacteria __Gammaproteobacteria __Pseudomonadales __Moraxellaceae __Acinetobacter __uncultured_bacterium'] |
| denovo11 | Bacteria __Proteobacteria __Gammaproteobacteria __Alteromonadales __Shewanellaceae __Shewanella'] |
| denovo303 | Bacteria __Proteobacteria __Gammaproteobacteria __Alteromonadales __Shewanellaceae __Shewanella __uncultured_bacterium'] |
| denovo18 | Bacteria __Proteobacteria __Gammaproteobacteria __Pseudomonadales __Moraxellaceae __Acinetobacter |

**The nasopharyngeal microbiota of feedlot cattle**

Devin B. Holman, Edouard Timsit, and Trevor W. Alexander

**Supplementary table S1.** OTUs found in 100% of entry samples (n=4) constituting the core microbiota.

**Supplementary table S2.** OTUs found in 100% of samples (n=4) taken after 60 days in the feedlot that constitute the core microbiota.

| OTU |  |
| --- | --- |
| denovo59 | Bacteria_Proteobacteria_Gammaproteobacteria __Pseudomonadales __Moraxellaceae __Psychrobacter |
| denovo339 | Bacteria __Proteobacteria __Gammaproteobacteria __Pseudomonadales __Pseudomonadaceae __Pseudomonas |
| denovo491 | Bacteria __Tenericutes __Mollicutes __Mycoplasmatales __Mycoplasmataceae __Mycoplasma |
| denovo42 | Bacteria __Proteobacteria __Gammaproteobacteria __Xanthomonadales __Xanthomonadaceae __Stenotrophomonas |
| denovo0 | Bacteria __Firmicutes __Bacilli __Lactobacillales __Carnobacteriaceae __Carnobacterium |
| denovo2 | Bacteria __Proteobacteria __Gammaproteobacteria __Pseudomonadales __Pseudomonadaceae __Pseudomonas |
| denovo3 | Bacteria __Firmicutes __Bacilli __Bacillales __Staphylococcaceae __Staphylococcus |
| denovo4 | Bacteria __Firmicutes __Bacilli __Bacillales __Staphylococcaceae __Staphylococcus |
| denovo8 | Bacteria __Tenericutes __Mollicutes __Mycoplasmatales __Mycoplasmataceae __Mycoplasma |
| denovo9 | Bacteria __Proteobacteria __Gammaproteobacteria __Pseudomonadales __Moraxellaceae __Psychrobacter |
| denovo135 | Unassigned |
| denovo1103 | Bacteria __Firmicutes __Bacilli __Bacillales __Staphylococcaceae __Staphylococcus |
| denovo1233 | Bacteria __Firmicutes __Bacilli __Bacillales __Staphylococcaceae __Staphylococcus |
| denovo17 | Bacteria __Proteobacteria __Gammaproteobacteria __Enterobacteriales __Enterobacteriaceae |
| denovo49 | Bacteria __Proteobacteria __Betaproteobacteria __Burkholderiales __Burkholderiaceae __Ralstonia |
| denovo307 | Bacteria __Firmicutes __Bacilli __Bacillales |

**Supplementary table S3.** Differentially abundant OTUs between feedlot entry and exit nasopharyngeal samples (n=4). False discovery rate (FDR) <0.05.

| **OTU Name** | **Day 0**  **(mean abundance)** | | **Day 60**  **(mean abundance)** | | **FDR** | **RDP Classifier Consensus Lineage** | | |
| --- | --- | --- | --- | --- | --- | --- | --- | --- |
| **More abundant in feedlot entry samples (day 0)** | | | | | | |  | |
| denovo11 | 266.25 | 7.25 | | 8.30E-67 | | Bacteria;__Proteobacteria;__Gammaproteobacteria;__Alteromonadales;__Shewanellaceae;__Shewanella | | |
| denovo0 | 304.25 | 17.75 | | 1.98E-66 | | Bacteria;__Firmicutes;__Bacilli;__Lactobacillales;__Carnobacteriaceae;__Carnobacterium;__uncultured_bacterium | | |
| denovo2 | 263.25 | 13.25 | | 1.29E-59 | | Bacteria;__Proteobacteria;__Gammaproteobacteria;__Pseudomonadales;__Pseudomonadaceae;__Pseudomonas;__uncultured_bacterium | | |
| denovo7 | 123.25 | 5.5 | | 1.98E-28 | | Bacteria;__Proteobacteria;__Gammaproteobacteria;__Pseudomonadales | | |
| denovo10 | 103.25 | 7.75 | | 7.59E-21 | | Bacteria;__Proteobacteria;__Gammaproteobacteria;__Pseudomonadales;__Moraxellaceae;__Acinetobacter;__uncultured_bacterium | | |
| denovo24 | 96.25 | 16.5 | | 3.05E-13 | | Bacteria;__Proteobacteria;__Gammaproteobacteria;__Enterobacteriales;__Enterobacteriaceae;__Enterobacter | | |
| denovo274 | 42.5 | 2.75 | | 9.99E-09 | | Bacteria;__Proteobacteria;__Gammaproteobacteria;__Pseudomonadales;__Pseudomonadaceae;__Pseudomonas;__uncultured_bacterium | | |
| denovo484 | 30.5 | 0.75 | | 1.83E-07 | | Bacteria;__Proteobacteria;__Gammaproteobacteria;__Pseudomonadales;__Pseudomonadaceae;__Pseudomonas;__uncultured_bacterium | | |
| denovo246 | 27.25 | 0.5 | | 7.09E-07 | | Bacteria;__Proteobacteria;__Gammaproteobacteria;__Pseudomonadales;__Moraxellaceae;__Acinetobacter;__uncultured_bacterium | | |
| denovo857 | 25.25 | 0.25 | | 1.02E-06 | | Bacteria;__Proteobacteria;__Gammaproteobacteria;__Alteromonadales;__Shewanellaceae;__Shewanella | | |
| denovo17 | 47.25 | 7.5 | | 1.02E-06 | | Bacteria;__Proteobacteria;__Gammaproteobacteria;__Enterobacteriales;__Enterobacteriaceae | | |
| denovo481 | 31.25 | 1.75 | | 1.10E-06 | | Bacteria;__Proteobacteria;__Gammaproteobacteria;__Alteromonadales;__Shewanellaceae;__Shewanella;__uncultured_Shewanella_sp. | | |
| denovo44 | 30.25 | 1.75 | | 1.98E-06 | | Bacteria;__Proteobacteria;__Gammaproteobacteria;__Pseudomonadales;__Moraxellaceae;__Acinetobacter;__uncultured_bacterium | | |
| denovo760 | 20.75 | 0.25 | | 1.83E-05 | | Bacteria;__Proteobacteria;__Gammaproteobacteria;__Alteromonadales;__Shewanellaceae;__Shewanella | | |
| denovo18 | 60.25 | 20 | | 0.000209 | | Bacteria;__Proteobacteria;__Gammaproteobacteria;__Pseudomonadales;__Moraxellaceae;__Acinetobacter;__uncultured_bacterium | | |
| denovo339 | 26.75 | 3.25 | | 0.000237 | | Bacteria;__Proteobacteria;__Gammaproteobacteria;__Pseudomonadales;__Pseudomonadaceae;__Pseudomonas;__uncultured_bacterium | | |
| denovo86 | 18.5 | 0.75 | | 0.000348 | | Bacteria;__Proteobacteria;__Gammaproteobacteria;__Pseudomonadales;__Pseudomonadaceae;__Pseudomonas;__uncultured_bacterium | | |
| denovo94 | 14.75 | 0.5 | | 0.00222 | | Bacteria;__Proteobacteria;__Gammaproteobacteria | | |
| denovo946 | 17.5 | 1.75 | | 0.00505 | | Bacteria;__Proteobacteria;__Gammaproteobacteria;__Pseudomonadales;__Moraxellaceae;__Acinetobacter;__uncultured_bacterium | | |
| denovo1104 | 12.25 | 0.25 | | 0.00559 | | Bacteria;__Proteobacteria;__Gammaproteobacteria;__Pseudomonadales;__Pseudomonadaceae;__Pseudomonas;__uncultured_bacterium | | |
| denovo936 | 16.25 | 1.5 | | 0.00633 | | Bacteria;__Proteobacteria;__Gammaproteobacteria;__Alteromonadales;__Shewanellaceae;__Shewanella | | |
| denovo1170 | 13 | 0.5 | | 0.00633 | | Bacteria;__Proteobacteria;__Gammaproteobacteria;__Alteromonadales;__Shewanellaceae;__Shewanella;__uncultured_Shewanella_sp. | | |
| denovo1159 | 17.5 | 2 | | 0.00657 | | Bacteria;__Proteobacteria;__Gammaproteobacteria;__Pseudomonadales;__Pseudomonadaceae;__Pseudomonas;__uncultured_bacterium | | |
| denovo407 | 14 | 1 | | 0.00955 | | Bacteria;__Proteobacteria;__Gammaproteobacteria;__Alteromonadales;__Shewanellaceae;__Shewanella | | |
| denovo990 | 12 | 0.75 | | 0.0205 | | Bacteria;__Proteobacteria;__Gammaproteobacteria;__Pseudomonadales;__Moraxellaceae;__Acinetobacter;__uncultured_bacterium | | |
| denovo209 | 17.75 | 3 | | 0.0212 | | Bacteria;__Proteobacteria;__Gammaproteobacteria;__Alteromonadales;__Shewanellaceae;__Shewanella | | |
| denovo97 | 10.5 | 0.5 | | 0.0290 | | Bacteria;__Proteobacteria;__Gammaproteobacteria;__Pseudomonadales;__Pseudomonadaceae;__Pseudomonas;__uncultured_bacterium | | |
| denovo40 | 20 | 4.75 | | 0.0431 | | Bacteria;__Proteobacteria;__Gammaproteobacteria;__Pseudomonadales;__Pseudomonadaceae;__Pseudomonas | | |
| denovo837 | 8.75 | 0.25 | | 0.0480 | | Bacteria;__Proteobacteria;__Gammaproteobacteria;__Alteromonadales;__Shewanellaceae;__Shewanella | | |
| **More abundant in 60 day feedlot samples** | | | |  | | | |  |
| denovo8 | 0.25 | 118.5 | | 2.03E-34 | | Bacteria;__Tenericutes;__Mollicutes;__Mycoplasmatales;__Mycoplasmataceae;__Mycoplasma | | |
| denovo14 | 4 | 109.25 | | 3.57E-26 | | Bacteria;__Proteobacteria;__Gammaproteobacteria;__Pseudomonadales;__Pseudomonadaceae;__Pseudomonas;__uncultured_bacterium | | |
| denovo9 | 34.25 | 163.5 | | 1.14E-19 | | Bacteria;__Proteobacteria;__Gammaproteobacteria;__Pseudomonadales;__Moraxellaceae;__Psychrobacter;__uncultured_bacterium | | |
| denovo23 | 0.25 | 21.5 | | 1.14E-05 | | Bacteria;__Actinobacteria;__Micrococcales;__Microbacteriaceae;__Zimmermannella;__Pseudoclavibacter;__Pseudoclavibacter_sp._G665 | | |
| denovo71 | 0.5 | 11 | | 0.0212 | | Bacteria;__Proteobacteria;__Betaproteobacteria;__Burkholderiales;__Comamonadaceae;__Comamonas;__uncultured_bacterium | | |
